# Valedictory Address, Delivered to the Medical Class in Lind University

**Published:** 1860-12

**Authors:** T. Deville


THE CHICAGO
MEDICAL EXAMINER

VOL. I. DECEMBER, 1860. No. 12

### ORIGINAL COMMUNICATIONS.

### VALEDICTORY ADDRESS,

Delivered to the Medical Class in Lind University,

By Prof. T. DEVILLE.

GENTLEMEN.—There are moments peculiarly felicitious in the lives of men who have devoted a considerable portion of their time to occupations which demand much patient labor, and where pecuniary recompense and honors are tardily dealt out, though had the same amount of energy been directed in channels of a different nature, they would most probably have lead to distinction and fortune.

I frankly avow at this moment that I feel vain and elated at the very flattering testimonial just presented me. Your appreciation of my qualities as a teacher, and your good wishes so kindly expressed for my future well-being and success, excites in my breast a feeling of honest pride and gratification which will be retained to the end of my existence.

None are so well qualified to judge the capabilities of the teacher, as the pupils who daily surround him ; pardon me the vanity of stating that this is another of the pleasing proofs, which it has been my good fortune to receive, that my labors have met the approval of those amongst whom I have found myself in the relation of an instructor.

In my introductory address last year, I distinctly disclaimed for myself any pretensions to eminence in my particular branch of science. I have again to reiterate it ; you will have seen that any success which is due to my teaching, has arisen rather from the energy and zeal with which I prosecute my duties, than from any special capabilities as a teacher. I love my calling, and nothing gives me greater pleasure than when I have been enabled to make some difficult point well understood by the class which I happen to have been instructing.

I feel deeply sensible of the very imperfect course of anatomy which I delivered in this school last year, for my circumstances sorely militated against me. To give a course of anatomy which may prove satisfactory to the teacher and of sound benefit to the student, I believe it should be of a more extended nature, the teacher should be aided by abundance of good preparations and diagrams, and above all, his whole energies should be concentrated on the responsible duties which the science peculiarly demands, his mind should be freed from the pressing anxieties of having to provide for his commonest necessities, and not exposed to causes which may depress his spirits, producing sometimes abstraction and incapacity from a hundred annoyances and disappointments.

I very much regret that my mission amongst you, has, in some respects, been unsuccessful, and that circumstances have obliged me to tender my resignation. I embarked my little all, the accumulated savings of a number of years, and contracted debts to provide myself with a suitable outfit for carrying on with credit a course of anatomy. Nothing would have induced me to abandon the post, and leave you, but the constant battle which I foresaw that I should for a long time be engaged in, to gain enough to meet the expenses of my moderate daily requirements. "The laborer is worthy of his reward," and when he cannot secure even a small recompense, he is driven by sheer necessity to seek it elsewhere. In the short space of twelve months, I am obliged to return to my native country, for my means will not permit me further to prolong my stay.

I do not enter into these details with the idea of a hacking blame to any individuals, for no one is responsible, it was my own act in coming out here. I thought that this would be a good point to meet with pecuniary success, that the opportunities which I had enjoyed in my profession would be duly appreciated, and remuneration sufficient to meet every moderate requisite immediately attained. I found on my arrival a great commercial depression, no money to be had, and but a few students. I realised in a moment the exact state of things, and began my career in this city with gloomy forebodings.

I must apologize for entering upon these purely personal details, and on which I ought perhaps to have been silent, but I felt that the explanation was due to my colleagues, so that no misinterpretation might get abroad as to the real causes which have led to my resignation. I am happy in being able to assure you that the relations which exist between myself and colleagues at the present time, are of a very friendly nature, I wish them sincerely success in their enterprise, that this department of the University may become the model institution of the North-West, and the graduates as a rule, recognized as men of superior intelligence by their brother practitioners.

Permit me here to offer a few remarks on medical education. I think preliminary education of great importance, more than seems to be insisted upon by the medical authorities of this country; I believe that a young farmer will find his progress in medicine very difficult, if he has not previously undergone disciplinary study.

The system of lecturing has been, and continues to be greatly abused, and one which cries loudly for reformation. This has been recognized in Europe, hence the evil has been much modified, and a better plan inaugurated.

I am proud to say that the Faculty of the Lind University is the first one, which in this country has acknowledged the truth of this proposition, and had commenced a system which possesses peculiar merits if rigidly carried out. To become connected with a highly intelligent body of professors who were engaged in the noble task of ameliorating the system of medical education was worthy the ambition of even a great man, and it was with the desire of aiding such a project by my humble efforts which powerfully induced me to accept the chair of anatomy amongst you.

When I was a student of medicine the vicious system of attendance upon six courses of lectures during the winter session and nearly as many in summer was in vogue, though the Faculty of medicine in King's College opposed it, and recommended that the students should attend not more than four daily, and so arranged the curriculum that the studies prolonged over another year, extra to the ordinary academical course. I well reccollect not having accorded with the recommendation, it being optional on the part of the student, as they could not enforce regulations which were in opposition to the ordinances of the examining boards. As I was a matriculated student, and the cost was no more, I thought I had better get through the prescribed course as quickly as possible, considering the system of lecturing as then practiced, a great bore and unmitigated evil, but in reviewing my student's career, I regret much that I had not accepted in full confidence the recommendation of my teachers. I had afterwards to sit about the earnest study of subjects on which I was imperfectly informed. My sin was visited not unfrequently on the head of a Mr. Deveulle, whose name so nearly approached my own, that we were often confounded, and errors arose notwithstanding the protestations of Mr. Deveulle. The Dean had made a remark against Mr. Deveulle's name, as having taken out six tickets contrary to the recommendation of the faculty, and in consequence strict examinations were to be inflicted.

I well reccollect one Monday morning when at the weekly *viva voce* examinations of the class, the Prof. of anatomy, in looking over the list of names came to that of Mr. Deveulle. Ah, says he, here's a gentleman who is I suppose a perfect walking encyclopœdia of anatomy and medical knowledge generally, as he is attending six courses of lectures. On examination poor Deveulle could not answer the question propounded, and got for his ignorance one of the notoriously caustic and pithy reprimands of the Prof. which elicited a war of laughter from nearly two hundred students.

I am glad to say that the curriculum first proposed by the faculty of King's College for the ordinary pass examinations, has been adopted by all the medical boards, and is now in full force.

The system of lecturing dates from the earliest period in the history of medicine when there was but few books; and almost the sole chance of instruction was by attending the lectures of celebrated professors scattered over Italy, Holland, Germany and France, one here, another there, thus entailing a long and expensive pilgrimage to the shrines of medical science, the avenues to which were difficult, and the laborers few.

Now, in this age of intelligence and progress we have abundance of books, enough to bewilder any student, and unless teaching be of a practical nature it loses much of its value. I do most solemnly protest against the system of six lectures, cliniques, and dissections per diem, as being utterly impossible on the part of the student to profit by such a course. If he gains knowledge it will be of such a superficial character as to be of no real value whatever, and if he pays particular attention to one branch of study, it will be to the detriment of others, all of which have a relative importance, and in either case, under such a system his medical education will be lamentably deficient.

And yet the improved system inaugurated in this University has been publicly denounced by the President of the neighboring college, notwithstanding all that has been said against the system pursued in his own school, by the highest representative of the profession in this country, the American Medical Association. I should like the pleasure of making a practical examination of such a set of students, after their second course, in Anatomy, Physiology, Pathology and Surgery. I would soon make them feel their real ignorance, and yet they receive the highest degree recognised in medicine, and go forth to the world pompously as educated physicians. The system is notorious and cannot be too loudly decried. My colleagues do not pretend that their own is anything like perfection, but they have *made one bold step* in the right direction, their system allows a little more breathing time for observation and reflection, and does not hurry them through the steam-mill at such a rapid rate. It is something to have accomplished this much, and entitles the faculty to the respect and confidence of every intelligent physician.

As preliminary to scientific studies an education in classics and mathematics is very desirable, it fits the mind for active work, besides being a sound embellishment. Again, before commencing medical studies proper, I think two full courses of six months, of a practical nature in natural Philosophy and Inorganic Chemistry, combined with two full summer courses in Botany and Natural History cannot be to highly estimated, if the student wishes to mark out for himself a distinguished position in the profession.

In Medicine, Surgery, Anatomy, Pathology, Chemistry, Physiology and Midwifery, the only rational manner of teaching these respective branches, is to teach them, as much as possible practically, at the bed-side, in the post-mortem theatre, dissecting room and labratory, aided by experiments, diagrams, moddles, preparations, &c.; otherwise the professor merely gives you so many words compiled from his own and others experience, which frequently is as quickly forgotten. This is very faulty, for the medical sciences are really those of observation and can only be taught effectively by way of demonstration.

I well remember attending a course of lectures at the Royal College of Surgeons in London, by Prof. Owen, on the homologies of the temporal bone. These lectures were very learned and scientific, so much so, that they entirely bewildered me, I was mentally obfusticated, until one day I had a simple demonstration on the subject of these lectures, and comprehended perfectly the essential leading points in less than half an hour.

Those branches of medicine which from their nature cannot be taught practically, I think it useless to exact the attendance of students on lectures; rather let the professor enjoin the use of a good text-book, make stated examinations on the subject, simplifying what is difficult to comprehend, and giving the result of his own observations as the occasion presents.

The system of examinations is one of great importance in medical education, and what I am glad to say is recognized by the faculty of this University. I think it might even be pushed further with advantage.

I have been in the habit for a number of years, as I have proceeded in a lecture, step by step, to ascertain if the students understand the point which has been demonstrated; in other words, whether I have made myself clear on the subject, adapted myself to their intelligence, and frequently I would drop on an unwary student, whom I thought perhaps to be at the time somewhat inattentive, with the happiest effects; a good moral discipline has been induced, and enthusiasm has prevailed, and what might otherwise have proved a dull lecture has been felt entertaining and useful; for once mistify a student and his interest in the lecture flags, he cares but little about it, and is glad to hear the bell summons the professor to shut up. Weekly *viva voce* and monthly written examinations are exceedingly useful, and even frequent examinations held amongst students themselves, is, I believe, of the highest benefit. This practical system may be taken as a fair outline of the one which has been gradually brought about, and is now employed in several of the best British schools.

I have given these few practical hints on medical education as the result of observation and my deep convictions. On the subject of education generally, and the difficulties which beset the medical student in the outset of his career I will not venture to enter, it has been so admirably treated by my talented and esteemed colleague, Prof Byford, at the recent Introductory Lecture, that I can but echo his deductions. His apt illustrations proved incontestably that our attainments depend much on our own individual energies, and confirmed the truth of the old adage, "whatever man has done, man may do." I advise you to ponder deeply over the sound counsel then given, and determine each of you to profit by it, in vigorous resolutions faithfully carried out in spite of the many obstacles you may have to contend with.

This brings to my recollection an era in my own history: I had finished the ordinary curriculum of medical education necessary for the position of a general practitioner. One day I took a walk and found myself at the foot of Vauxhall Bridge, London, in a neighborhood which was at that time comparatively free from noise and bustle. I sat down on a large stone, of which there were groups lying about for building purposes, and soon became buried in deep thought. The question presented to my mind for solution was one of the highest importance: it was no other than my future destiny. Shall I now commence general practice, or shall I prepare myself to take a creditable position in the profession? I determined on the latter course. It is said we act sometimes in the spirit of contrariety, and so I was judged for a long time, for my friends desired me to commence practice immediately. I had exhausted all my means, upwards of seven thousand dollars, and now, if I persisted staying in London I could hope but for little aid from home, my friends would gladly have assisted me in the early years of private practice, but spending longer time and money in study was considered by them as utter folly. I revolved over in my mind the necessary steps by which I could ultimately gain reputation, and the more I thought on the matter, the immensity of the difficulties which would beset me almost appalled me. I loved anatomy, and determined that I would make its study the basis of my future success.

I was unprotected by the friendly hand of any distinguished man, to watch over and push my interests, and when I contemplated the many great intellects which served me as models, towering up in such colossal grandeur, I thought it great presumption on my part for ever daring to attempt the task. Nevertheless, I set about the work in right earnest; I pledged my fine gold watch to raise money for dissecting materials, and obliged to earn my own living; every spare hour was consecrated to the one great object, the pursuit of anatomy, and thus it has gone on from days to weeks, from weeks to months, and from months to years; and though I many times bruised my shins against opposing rocks, which obliged me for the time to abandon the path, still I soon regained it, and urged onwards with ever-recurring ardour, and now I begin to feel I have made some progress towards the summit of my ambition. Though the mountain top is yet clouded, the vapor is thinner, and gradually unveiling, I think I descry the ultimate boundaries. Still there is much to accomplish, and I will not now abandon the effort; I feel it to be the one great object with which my life is identified, and to which I will consecrate my best energies; if I fail, I shall have at least the satisfaction that my labors were creditable.

My attention has been drawn to an article by Prof. Brainard in the *Chicago Medical Journal*, for this month, entitled "Notes relating to the extirpation of the Parotid Gland." It contains remarks which I cannot allow to pass unnoticed. As to the arguments and so-called facts brought forward, I shall take an early opportunity to prove their entire fallacy, and though I cannot raise my voice, rest assured, gentlemen, I will not fail to wield my pen with vigorous effort, and from a quarter where evidence and materials will not be wanting to aid me.

Though scarce a notice is made of me in the article alluded to, it is worthy of comment that it appears eight months after the criticism, when it was perhaps fondly hoped "la diable" was out of harm's way; but you may depend that once having entered on a discussion, it is a matter of difficnlty to beat me, for I never engage on a subject unless I am deeply convinced, from undeniable proofs, that I have truth on my side.

Prof. B. tries to get up a cry of persecution, and insinuates that the Faculty of this University are trying to hunt him down. This, I believe, to say the least of it, to be a greivous error under which he is laboring, and is far from the truth. I know well that I had a difficulty at the time with my colleagues on the propriety of this attack, and was remonstrated with; I am not discreet, never was, or care to be, so long as I am sustained by weighty and sensible reasons. I claim for myself independent action, and entered on the discussion entirely on my own responsibility. I must here solemnly protest, in justice to my colleagues, againt the assertion of Prof. Brainard, that the Faculty are determined to make war on him, or that they had any connection whatever with my critical lecture. It is the feeling, perhaps, that the cap fits well on the other side, which has given birth to such an absurdity. These petty jealousies and rivalries are the failings, and, to a certain extent, the appendage of humanity, which ought to be kept within bounds, or they will prove an endless source of misery to the

2

individual who displays such a weakness. For my own part, I feel none towards Prof. Brainard or his colleagues; I should extend to them the same cordial feeling I do towards my own associates, if I had been permitted. True science is cosmopolitan, and merit is recognised wherever it is found by the votary of science however humble he may be. I stand up here to proclaim to you that in my opinion Prof. Brainard is infinitely superior to the great bulk of men around him, and his attainments are of a creditable nature. In justice he deserves commendation for what he has done in surgery; yet truth must be upheld, if he promulgates errors which are in opposition to the teachings of anatomy and the experience of those eminent professors who have so strenuously denounced them, whose brilliant talents I revere, from having so often listened with great profit from their own lips, and to whom I am indebted for much of the little knowledge I possess.

I will now briefly enter on the reasons which called forth on my part a strong protest and exposure of the article written by Prof. Brainard, "on the extirpation of the Parotid Gland!" I had previously taught the impropriety of this operation on the ground of its difficulty; but I never spoke of its utter impossibility. In this opinion I am supported by the highest anatomical authorities, whose unqualified testimony cannot be questioned.

I knew the opinion of the three most celebrated operating surgeons of modern times, compared to whom Prof. Brainard is a little child not yet emerged from his swaddling clothes, who affirm most positively and unequivocally that they have never extirpated the parotid gland, or seen any disease thereof, the so-called diseases were really enlargement or degeneration of the lymphatic glands in that region, and the operations performed were wrongly construed.

In his late article he brings forward ninety cases of the parotid extirpation, "a-la Brainard," and yet three of the greatest surgeons *have never seen one.*

I like the style of my old teacher, Prof. Fergusson, who begins his remarks on the subject by putting down these exploded errors in terse terms,—" Twenty years ago," says he, " it was the custom to speak of extirpation of the parotid," &c., &c. And here comes an important duty—it is that of being his champion when his authority is ignored on a subject on which he has spoken out so positively. I should smile at the derisive sneers of the whole medical men of Chicago, even if every one estimated me as an ignorant fool, so long as I feel that I have the confidence of yourselves and my colleagues to sustain me. I feel assured that you would be as so many valient hearts to defend me right manfully from the machinations of the libeller.

Not one argument which I brought forward has been fairly met by Prof. Brainard, and as to his assertion that any practitioner can detect me in a "gross error," I have only to say, gentlemen, wait a little longer.

On referring back to the experience of last winter, there was much to gratify me and counterbalance the many discouraging circumstances under which I labored. We got up quite a respectable amount of enthusiasm and work on anatomy; many nights of the week found you, my dear fellows, trudging for miles in some cases, in spite of frost and snow, wind and rain, to my rooms or the college. We had glorious times, a kind of anatomical jubilees that made me forget when amongst you, the sorrows which lay so heavily on my heart. Yes, my old pupils, I shall always have pleasant reminiscences of my past career in Chicago, when I associate it with your attention and respectful homage.

I have fallen crippled, but I feel assured by your testimonial this day that I have not fallen ingloriously; I hope soon again to regain my feet, and mount slowly the hill of science. My worthy and revered colleague, Prof. Hollister, who is already so favorably known to you, has succeeded me, and I feel great pleasure in bearing my humble testimony to his energy and ability, and which are so gracefully combined with the principles which make the true gentleman and christain. In his hands I resign the trust, confident that he will ably lead you on; and I hope I may live to see the day when this institution will have attained a high degree of reputation. I feel myself identified with it, all my hopes in life have been centered here, and I shall ever entertain a lively interest in its progress.

Wherever you may be, rest assured I will not forget the individual members of the little band whom I had the pleasure to direct, and who cheered and animated me by their kindness and devotion. My dear old pupils, I will cherish your memories.

And now I come to the last sad task, to bid you, one and all, affectionately, Farewell.

